# Challenges and needs of nurse researchers on research publication in health universities of Karnataka, India: a cross-sectional survey

**DOI:** 10.1177/17449871251347840

**Published:** 2025-08-14

**Authors:** Remya U Rajendran, Mamatha Shivananda Pai, Baby S Nayak, Shalini G Nayak, Judith Angelitta Noronha, Santhosh Krishnan Venkata, Vimala Ramoo, Accamma Oommen, Vishnu Renjith

**Affiliations:** Research Scholar, Department of Child Health Nursing, Manipal College of Nursing, Manipal Academy of Higher Education, Manipal, Karnataka, India; Professor, Department of Child Health Nursing, Manipal College of Nursing, Manipal Academy of Higher Education, Manipal, Karnataka, India; Professor & HOD, Department of Child Health Nursing, Manipal College of Nursing, Manipal Academy of Higher Education, Manipal, Karnataka, India; Assistant Professor, Department of Medical-Surgical Nursing, Manipal College of Nursing, Manipal Academy of Higher Education, Manipal, Karnataka, India; Professor & Dean, Department of Obstetrics & Gynaecological Nursing, Manipal College of Nursing, Manipal Academy of Higher Education, Manipal, Karnataka, India; Professor, Department of Instrumentation & Control Engineering, Manipal Institute of Technology, Manipal Academy of Higher Education, Manipal, Karnataka, India; Associate Professor (Research Coordinator), Department of Nursing Science, Faculty of Medicine, Universiti Malaya, Kuala Lumpur, Malaysia; Associate Professor, Head of Nursing, Torrens University, Australia; Lecturer/Programme Director, School of Nursing & Midwifery, Royal College of Surgeons in Ireland, Dublin, Ireland

**Keywords:** Mentorship, Nursing faculty, Nursing research, Publishing, Support of research, Writing

## Abstract

**Background:**

Effective dissemination of scientific knowledge through publication is vital for advancing nursing and healthcare. However, nurse researchers often encounter challenges that impede their ability to publish in reputable journals.

**Aim:**

To identify the perceived challenges and support needs related to research publication among nurse researchers at health universities across Karnataka, India.

**Methods:**

A multicentre web-based cross-sectional survey was conducted from January to May 2024 across 33 nursing institutes with 319 nurse researchers.

**Results:**

Of the 319 respondents, 58.6% had published papers, but only 15% had published in indexed journals. The most frequently reported barriers to publication were lack of time (53.3%), funding constraints (42.9%) and difficulty in initiating writing (27.9%). Limited writing skills were significantly associated with academic designation (χ² = 11.9, *p* = 0.003) and difficulty starting writing (χ² = 15.9, *p* < 0.001). A substantial majority expressed a need for technical support in areas such as manuscript formatting (87.9%), submission (85%) and responding to reviewers (84.6%). The primary motivations for publication included career advancement (67.7%) and meeting institutional requirements (50.2%).

**Conclusion:**

This study underscores the pressing need for targeted interventions, including structured training, technical assistance and mentorship, to support nurse researchers in overcoming publication challenges and enhancing scholarly productivity.

## Introduction

Publications are vital in translating scientific evidence into accessible knowledge, profoundly impacting the research community and public understanding of healthcare advancements (Johann et al., 2024; Kaur, 2013). The primary focus for disseminating research findings is professional and scientific audiences, so prioritising strategies for sharing these findings with a broader audience is essential (Melvin et al., 2020; Morrato et al., 2013). For nursing professionals, writing for publication in reputable journals is essential for advancing healthcare practices and ensuring that nurses remain informed about the latest research (Edwards, 2015; Sánchez-Gómez et al., 2023). The effective dissemination of research findings empowers both public and fellow researchers, fostering a culture of inquiry and enabling further advancements in healthcare (Barría, 2022).

However, despite the crucial need for dissemination, significant barriers hinder researchers from successfully publishing their work, particularly in health sciences. The challenges primarily stem from a lack of writing skills, inadequate mentorship, limited access to research facilities and insufficient financial resources (Alotaibi, 2023; Kadikilo et al., 2024). Studies have shown that many institutions are underperforming in their publication outputs. According to an analysis of 579 Indian medical colleges’ and hospitals’ research publications, only 25 (4.3%) institutions generated more than 100 papers annually, with over half (57.3%) not producing any publications in a decade(Tullu and Karande, 2016). India’s research output in top global journals remains low, with only 15.8% of publications featured in the top 10 journals, underscoring the urgent need to enhance research quality, international collaboration, and academic support systems (Chopra, 2019).

Furthermore, institutional and individual barriers often impede effective communication and the ability to publish. Postgraduate faculty and PhD scholars face significant organisational hurdles and personal challenges, such as limited writing experience (Dahal et al., 2022). Many challenges exist for researchers in publishing academic papers, including a lack of skill in manuscript writing and submission, and the selection of journals, which are identified as the major barriers to research publication (Arshabayeva et al., 2024; Paiva et al., 2017). A cross-sectional study of nursing faculty in India highlighted that fewer than 7% of the studies published in national journals revealed that financial support and inadequate learning resources were prominent obstacles (Chandekar, 2021).

Various international studies highlight common challenges to publication, including time constraints, inadequate training in research methodology, and financial difficulties. For example, medical interns in Saudi Arabia identified a lack of training and time as key barriers (Hakami, 2023). At the same time, PhD students in Malaysia reported internal and external obstacles, ranging from low English proficiency to unfavourable review outcomes and difficulties coordinating with co-authors (Diat et al., 2019). Similar issues were reported in Nigeria and France, where factors such as poor research facilities and limited mentorship hindered publication efforts (Duracinsky et al., 2017; Okoduwa et al., 2018). Despite researchers’ efforts to conduct novel studies, high rejection rates and competition limit publication in high-quality journals, with a lack of confidence in writing being a key barrier (Ali, 2010; Chavda et al., 2021).

To address these challenges, implementing comprehensive support systems such as scientific writing workshops and mentorship programmes is essential. By fostering a research culture through targeted training and resource allocation, institutions can significantly enhance publication outcomes and the overall impact of healthcare research (Yancey, 2016). Ultimately, bridging the gap between evidence generation and practical application in nursing research is crucial for improving healthcare outcomes (Hakami, 2023; Hanneke and Link, 2019).

Several studies have noted the challenges faced by nurse researchers, but there has been no comprehensive investigation into these issues, and many studies lack strong methodologies. While various challenges are recognised, it is essential to explore these difficulties, specifically within the context of nursing researchers at health universities in a structured way. This survey identifies the specific challenges and needs perceived by nurse researchers in India regarding research publication and can provide targeted insights to develop tailored interventions, such as mentorship programmes, writing support and better resource allocation.

## Methods

### Aim

The study aimed to identify nurse researchers’ perceived challenges and needs in research publications. This paper has been reported in accordance with the guidelines outlined in the Checklist for Reporting of Survey Studies (CROSS) to ensure comprehensive and transparent reporting of the survey findings (Sharma et al., 2021).

### Design and setting

This study employed a descriptive cross-sectional design and was conducted among nurse researchers from nursing colleges affiliated with public and private health universities in Karnataka, India, between January 2024 and May 2024. These institutions serve as hubs for nursing practice, research, and education. The numerous nursing colleges highlight the extensive and diverse nursing education system in Karnataka, offering many opportunities for sampling and data collection.

### Sample and sample size

The participants were selected on the basis of their qualifications (postgraduate or above in nursing) and their experience in conducting research. Individuals without research experience were excluded from the study.

A specific formula was used to determine the appropriate sample size:

*n* = $\frac{{\left(Z_{1-\frac{\propto }{2}}\right)}^{2}pq}{{\left(d\right)}^{2}}$ at a significance level of α = 0.05, the critical value of 1.96 was selected, corresponding to a 95% confidence interval. The key components in the calculation included p, the expected proportion of the population; *q*, the complement of *p* calculated as (1−*p*); and *d*, the allowable error, which was set at 5% of *p*. The required sample size was calculated as 317 participants. A total of 990 participants from 33 nursing institutes were invited to participate in the survey, and 322 completed the questionnaire within the given time frame. However, responses from three participants were removed because of incomplete details. Data were ultimately collected from 319 nurse researchers.

### Instruments

*Demographic Proforma*: A 12-item structured questionnaire was developed by the researcher to evaluate the sociopersonal characteristics of nurse researchers. The tool was validated by a panel of five to seven experts, and the content validity index (CVI-1) was calculated.

DIAzePAM Survey Questionnaire: The DIAzePAM (Difficultés des Auteurs à la Publication d’Articles Médicaux) [translation: challenges for authors in publishing medical articles] survey questionnaire was developed by French researchers. An English version of the questionnaire is available in the public domain and can be used without copyright restrictions. No formal permission is required for its use, reproduction or adaptation. This tool was used to assess the challenges faced by nurse researchers during the publication process (Duracinsky et al., 2017). This semi-structured questionnaire comprises 15 main items with subitems, resulting in 39 items. The DIAzePAM tool demonstrated strong psychometric properties, with a content validity index, CVI-1 and a reliability score (Cronbach’s alpha) of 0.786. It provided insights into critical areas such as medical writing experience, encountered difficulties, respondents’ positions regarding paper publication, and the need for external support. Additionally, the questionnaire captured nonidentifying sociodemographic and occupational characteristics, which helped provide context for the findings.

Both instruments were pretested online by 10 nurse researchers to confirm feasibility and clarity before full implementation.

### Survey administration

The survey was conducted as a web-based study via a single-time administration approach. Data collection was initiated by sending a formal request email to the head of the institution (HOI) of 33 nursing institutes, detailing the study and the inclusion criteria. The HOI then provided a list of faculty members from their institution who met the eligibility criteria and their email addresses. A total of 990 faculty members were invited through the Google Forms platform, and the questionnaire was distributed to nurse researchers via their registered email addresses. The functionality was tested on Google Chrome for a smooth experience. The questionnaire was available online for 15 weeks, from 31 January 2024 to 21 May 2024, during which two reminder emails were sent at 1-week intervals to encourage participation. Google Form employs advanced encryption protocols to protect data during transmission and helps safeguard responses against unauthorised access.

### Ethical considerations

Ethical approval for the study was obtained from the Institutional Ethics Committee (IEC) (Approval No. IEC/29/2023), which was granted on 5 July 2023. The study adhered to strict ethical standards to ensure the confidentiality of participants' information. Participants were informed of their right to withdraw from the study at any time without any repercussions. Electronic informed consent was obtained before participation, and their eligibility as faculty members at health sciences universities in Karnataka was verified. This ensured transparency and compliance with ethical guidelines throughout the research process.

### Statistical analysis

The data were entered, coded and analysed via Jamovi software 2.4.7 (the Jamovi project, *School of Psychological Sciences, University of Newcastle, Australia*) because of its user-friendly interface and compatibility with the research objectives. Descriptive statistics (frequency, percentage, mean, median and standard deviation) were used for analysis. The chi-square test was applied to investigate associations between demographic characteristics and the domains of needs and challenges, with a significance level set at *p* < 0.05.

## Results

A total of 322 nurse researchers completed the Demographic Proforma and the DIAzePAM questionnaire. Ultimately, 319 participants met the eligibility criteria and were included in the survey.

The demographic and professional characteristics of the nurse researchers, summarised in Table 1, show that among the 319 participants, 50.1% were middle-aged (45–60 years), 81.8% were female and the majority held an MSc in nursing (79.6%), with 53.6% having 6–15 years of professional experience. With respect to research background, 38.9% had <5 years of experience, 70.2% were not part of any research group, only 35.1% had formal training in publication, and 58.6% had published papers.

**Table 1.** Demographic and professional characteristics of the nurse researchers (*N* = 319).

Demographic characteristics	*N* (%)*	*M* (SD)
Age in years		37.8 (7.18)
Young adult (25–44)	139 (43.6)	
Middle-aged (45–60)	160 (50.1)	
Older adult (61–75)	20 (6.3)	
Gender		
Male	58 (18.2)	
Female	261 (81.8)	
Educational qualification		
PhD (N)	63 (19.8)	
MSc (N)	254 (79.6)	
MPhil (N)	2 (0.6)	
Work Experience in years		
⩽5	81 (25.4)	
6–15	171 (53.6)	11.1 (7.06)
16–25	56 (17.6)	
>25	11 (3.4)	
Job title		
Professor Associate professor	84 (26.3) 56 (17.6)	
Assistant professor	103 (32.2)	
Lecturer	76 (23.8)	
Specialty		
Medical-surgical nursing	93 (29.2)	
Child health nursing	68 (21.3)	
OBG nursing	70 (21.9)	
Community health nursing	37 (11.6)	
Mental health nursing	51 (16)	
Involvement in scientific research (in years)		
Less than 5	124 (38.9)	7.27 (5.63)
5–10	115 (36.1)	
More than 10	80 (25.1)	
Member of research collaborative group		
Yes	95 (29.8)	
No	224 (70.2)	
Received any training on publication		
Yes	112 (35.1)	
No	207 (64.9)	
Did you publish any papers?		
Yes	187 (58.6)	
No	132 (41.4)	
If yes (*n* = 187)		
Mention the details of publication		
Indexed	28 (15)	
Non-indexed	159 (85)	
Number of publications(*n* = 187)		
<5	109 (58.3)	4.06 (7.70)
5-10	47 (25.1)	
More than 10	31 (16.6)	

SD: standard deviation.

* The frequency (*N*) and percentage (%) were calculated from the total number of respondents (*n* = 319)

### Details on publication and authorship

Among the 187 published participants, only 28 (15%) published their papers in indexed journals. Conversely, 159 participants (85%) published in nonindexed journals, indicating a preference or tendency towards journals that may not be recognised as indexed journals, and a significant portion of the participants (58.3%) had fewer than 5 publications. With respect to previous publications, the findings reveal that over half of the respondents (52.9%) had served as primary authors for at least one paper in the past 2 years. Additionally, 43.9% contributed as the last author, whereas 42.2% were in the role of the corresponding author during the same period, highlighting active engagement in various authorship roles.

### Barriers to nurse researchers in publishing papers

**Figure 1.**
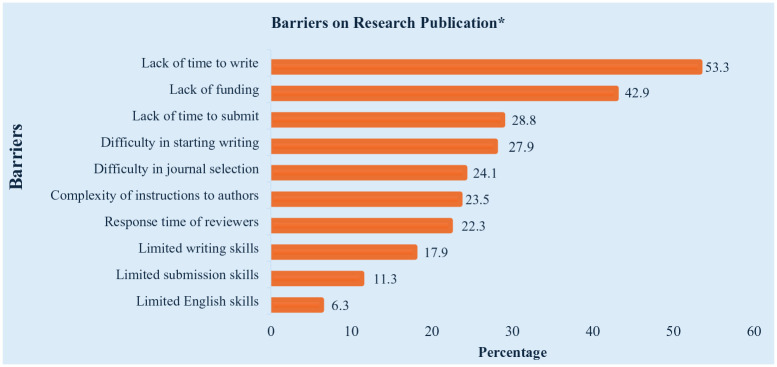
Barriers reported by nurse researchers in publications (*N* = 319). Responses are presented as percentages. *More than one response.

The most common barrier to publishing was lack of time to write (53.3%), followed by lack of funding for publication fees (42.9%) and difficulty starting writing (27.9%). Other challenges included submission delays (28.8%), complex author guidelines (23.5%) and reviewer response times (22.3%). Limited writing (17.9%) and English skills (6.3%), as well as coordination issues with co-authors (12.9%), were also reported. A small percentage (2.8%) cited other barriers (see Figure 1).

Age, gender, work experience, and research involvement were not significantly associated with limited writing skills. However, job title/designation (χ² = 11.9, *p* = 0.003) was a key factor, with higher designations linked to better writing skills. Difficulty in starting writing (χ² = 15.9, *p* < 0.001) was strongly associated, emphasising it as a major barrier. Training on publication (χ² = 3.82, *p* = 0.051) indicates that the length of professional or research experience does not influence writing skills (See Table 2).

**Table 2.** Factors associated with self-reported limited skills in writing (*N* = 319).

Variables	Limited writing skills		χ^2^	*p*-Value
	Yes (*n* = 56)	No (*n* = 263)		
Age (years)			0.508^a^	0.476
24–43	22	117		
44-62	34	146		
Gender			0.0975^a^	0.755
Female	45	216		
Male	11	47		
Job title/designation			11.9^a^	0.003*
MSc nursing	39	215		
PhD nursing	15	48		
MPhil nursing	2	0		
Work experience (years)			2.58^a^	0.275
<5	10	71		
6–15	35	136		
16–25	11	56		
Involvement in scientific research (years)			1.59^a^	0.453
<5	18	106		
5–10	21	94		
More than 10	17	63		
Received training on publication			3.82^a^	0.051*
Yes	26	86		
No	30	177		
Difficulty starting writing			15.9^a^	<0.001*
Yes	28	62		
No	28	201		
Lack of time to write			1.87^a^	0.171
Yes	35	138		
No	21	125		

a Pearson’s chi-square test was used to find the association.

* Significant at 0.05 level.

### Perceived need for support in research publication

Among the 319 participants, 87% (*n* = 279) reported the need for technical support. The highest need was reported for formatting manuscripts (87.9%), followed by manuscript submission (85%), responding to reviewers (84.6%) and manuscript writing (82.4%). Support for English editing (77.8%), preparing tables and figures (74.2%) and journal selection (67.1%) were also significant. This indicates a widespread demand for technical support services to aid researchers in various aspects of the publication process.

With respect to funding support for various aspects of publishing, most (66.7%) indicated a need for support to cover publication fees, whereas 29.4% required assistance with open-access fees. Support for medical writing was needed by 15.4%, and 11.5% sought help with translation or English editing. Interestingly, 16.1% of the respondents reported no need for funding support.

### Journal selection criteria and preferred publication model

The participants were asked about the criteria they considered when selecting a journal for publication. The top criteria for journal selection were the impact factor (53.9%), acceptance rate (48%) and publication speed (46.4%). Open access was preferred by 67.1% of the respondents, whereas 20.4% had no preference. Less common factors included journal ranking (28.8%), the ability to suggest reviewers (14.1%) and topic relevance (13.5%; Table 3).

**Table 3.** Journal selection criteria and preference of journal model (*N* = 319).

Journal selection and preference	(*N*)	(%)
Journal selection criteria*		
Acceptance rate	153	48
Impact factor	172	53.9
Publication speed	148	46.4
Degree of specialisation	128	40.1
Topic already accepted by the journal	43	13.5
High-rank classified journals	92	28.8
Open-access journal	112	35.1
Acquaintance with one of the editors	12	3.8
Opportunity to suggest reviewers	45	14.1
Journal model		
Open access	214	67.1
Classic model	40	12.5
No preference	65	20.4

* More than one response.

Responses are presented in frequency (*N*) and percentage (%).

Motivation for publication.

The participants identified various motivations for pursuing publication, with the most frequently cited reason being career advancement/promotion (67.7%), followed by mandatory requirements (50.2%) and influencing practice changes (39.5%). Other reasons included peer recognition (31.7%), institutional pressure (25.3%) and the novelty of research (23.5%). Less common factors were information dissemination (22.9%), maintaining study records (19.7%) and financial incentives (15%; Table 4).

**Table 4.** Motivation and need for publication as perceived by nurse researchers (*N* = 319).

Reason for publication*	(*N*)	(%)
Primacy of novelty	75	23.5
Educational role/mandatory requirement	160	50.2
Changes in practice	126	39.5
Keeping an official record of study results	63	19.7
Peer recognition/reputation	101	31.7
Career advancement/promotion	216	67.7
Financial interest/incentives from the institution	48	15
Pressure from medical hierarchy	23	7.2
Information dissemination	73	22.9
Pressure from institutional hierarchy	81	25.3
Confirming previous studies	30	9.4

Responses are presented in frequency and percentage.

N: Frequency; %: Percentage.

* More than one response.

## Discussion

Publishing is a critical yet challenging aspect of the research process. This study investigated the significant challenges encountered by nurse researchers in scientific publishing. A survey of 319 nurse researchers from private and public health sciences universities across Karnataka highlights critical challenges that must be addressed to increase academic contributions in the field. The findings use a cross-sectional survey to inform strategies that support faculty in overcoming these hurdles and advancing their scholarly work.

The study highlights that time constraints and insufficient funding remain the most significant barriers to scientific writing, aligning with previous research (Majid et al., 2022). Unlike earlier studies, our findings emphasise that difficulty in initiating writing and navigating submission guidelines are equally critical. Additionally, barriers such as limited English proficiency, unfamiliarity with writing conventions, high costs of accessing online resources, challenges in avoiding plagiarism and difficulty in selecting appropriate topics further hinder scientific writing (Alkhuzaee et al., 2019). Coordination with co-authors and fear of negative results further compound these challenges, reinforcing the need for an inclusive and supportive academic environment through improved mentorship and institutional practices (Shivananda et al., 2024).

A notable finding is the widespread demand for technical support in manuscript preparation. Researchers struggle with essential tasks, including responding to reviewers, formatting manuscripts and resubmitting revised papers. While peer review remains a cornerstone of academic publishing, our findings suggest that early exposure to the peer-review process at the institutional level may help inculcate critical writing and revision skills, making manuscript preparation less daunting (Candal-Pedreira et al., 2023).

Journal selection remains another area requiring strategic intervention. While the impact factor and acceptance rates are key considerations, our findings highlight a strong preference for open-access publishing, emphasising the need for institutional funding to support publication fees. When selecting a journal for publication, researchers should consider journals used by peers, assess their scientific rigour and editorial quality by reviewing past publications; and evaluate indicators such as aims, editorial board, indexing, the peer-review process, reputation, and author policies (Remya et al., 2024; Suiter and Sarli, 2019). Enhanced training in scientific writing, manuscript preparation, and journal selection has been proposed to increase publishing ability (Pittman et al., 2017).

Additionally, participants identified career advancement and mandatory academic requirements as primary motivators for publication, with fewer researchers emphasising knowledge dissemination or practice changes. This underscores the need for a cultural shift that values research beyond career incentives, encouraging publications that contribute meaningfully to nursing practice. Nursing institutions are strongly advised to act on this matter by establishing structured research programmes that incorporate mentorship, funding support, and protected writing time - which are essential.

To address these issues, institutions must adopt a multifaceted approach that includes writing workshops, peer-review mechanisms and early integration of scientific writing training at the student level. Focusing on scientific writing, peer-review training and journal selection strategies can significantly enhance researchers’ publishing success (Darawad et al., 2018). These workshops provide structured guidance on manuscript preparation, effective communication of research findings and adherence to journal submission guidelines (Wortman-Wunder and Wefes, 2020). Moreover, writing workshops create an environment where participants can receive immediate feedback, thus reducing common anxieties associated with scientific writing. By offering hands-on training, expert mentoring and peer discussions, workshops help participants refine their writing skills, enhance the confidence of nursing personnel and develop a deeper understanding of the publication process (Bellicoso et al., 2022). Programmes such as a mentored writing programme provide researchers with the necessary skills to refine and publish their work, thereby maximising research impact.

In addition to workshops, peer review plays a crucial role in improving scientific writing skills. A faculty development workshop with peer-writing groups enhanced writing productivity, increased manuscript submission, and improved writing quality through structured peer review and valuable feedback in medical education (Steinert et al., 2008). Engaging in peer review and receiving feedback significantly enhances health sciences students’ scientific literacy and writing skills. Through multiple writing assignments and revisions based on peer and instructor feedback, students reported improved knowledge, skills, and attitudes towards scientific writing (Geithner and Pollastro, 2016). Institutional initiatives encouraging faculty and students to participate in peer-review groups can facilitate constructive discussions, promote collaborative learning, and enhance manuscript quality before formal journal submission.

To build long-term capacity in scientific writing, it is essential to integrate these skills into academic curricula at the student level. Early exposure to research writing, literature reviews, and publication ethics can help students develop confidence and competence in scholarly communication. Universities should incorporate structured scientific writing courses, assign writing-intensive projects, and encourage students to engage in journal clubs, where they critically analyse published research. Additionally, mentorship programmes that pair students with experienced researchers can provide continuous support, guiding them through the writing and publication process from an early stage. Implementing best practice models for writing resources within health departments and demonstrating successful organisational cultures that encourage writing and publishing can significantly increase our efforts (Pittman et al., 2017).

### Limitations

The DIAzePAM Survey Questionnaire has several limitations that could affect the validity of its findings. Primarily, the reliance on closed-ended questions restricts the depth of responses, potentially omitting valuable insights into participants’ experiences and barriers related to research publication. Institutional policies regarding publication may also discourage healthcare professionals from engaging in research, leading to gaps in knowledge and practice. These factors underscore the need for caution in interpreting the results, suggesting that future surveys should incorporate open-ended questions to better understand the complexities surrounding DIAzePAM questionnaire usage. Overall, although the survey offers quantitative data, it highlights the importance of qualitative insights in effectively informing healthcare practices.

## Conclusion

In conclusion, the challenges faced by nurse researchers in scientific publishing underscore the need for comprehensive support systems to increase academic contributions in the field. Challenges such as time constraints, inadequate writing skills, and financial limitations must be addressed through mentorship programmes, workshops, and clearer resources to encourage a more inclusive and productive publishing environment. By fostering a culture that values diverse research outputs and providing targeted training in writing and journal selection, the academic community can empower researchers to overcome obstacles, thereby advancing knowledge dissemination and enhancing the overall quality of scientific literature.

**Key points for policy, practice and research**

- Addressing the publishing challenges that nurse researchers face can help institutions develop strategies to increase confidence and skills in scientific writing.
- Providing training in navigating publication processes will empower nursing researchers to make informed decisions regarding where to submit their work, thereby increasing publication success rates.
- Health and social care policies must include provisions for allocating funding and time for research activities, promoting a culture of inquiry and encouraging nurse researchers to engage actively in scholarly pursuits.
- Future research should evaluate the effectiveness of support initiatives and mentorship in overcoming publishing barriers and explore the impact of increased research output on nursing practices and healthcare outcomes.
